# Early life adverse exposures in irritable bowel syndrome: new insights and opportunities

**DOI:** 10.3389/fped.2023.1241801

**Published:** 2023-09-05

**Authors:** Guo Qiong Zhou, Meng Jie Huang, Xin Yu, Na Na Zhang, Shan Tao, Ming Zhang

**Affiliations:** Department of General Practice, Honghui Hospital, Xi'an Jiaotong University, Xi’an, China

**Keywords:** microbiota, early life, maternal, children, intrapartum, irritable bowel syndrome

## Abstract

Irritable bowel syndrome (IBS) is a prevalent functional gastrointestinal disorder worldwide. Extensive research has identified multiple factors contributing to its development, including genetic predisposition, chronic infection, gut dysbiosis, aberrant serotonin metabolism, and brain dysfunction. Recent studies have emphasized the critical role of the early life stage as a susceptibility window for IBS. Current evidence suggests that diet can heighten the risk of IBS in offspring by influencing the microbiota composition, intestinal epithelium structure, gene expression, and brain-gut axis. The use of antibiotics during pregnancy and the neonatal period disrupts the normal gut microbiota structure, aligning it with the characteristics observed in IBS patients. Additionally, early life stress impacts susceptibility to IBS by modulating TLR4, NK1, and the hypothalamic-pituitary-adrenal (HPA) axis while compromising the offspring's immune system. Formula feeding facilitates the colonization of pathogenic bacteria in the intestines, concurrently reducing the presence of probiotics. This disruption of the Th1 and Th2 cell balance in the immune system weakens the intestinal epithelial barrier. Furthermore, studies suggest that delivery mode influences the occurrence of IBS by altering the composition of gut microbes. This review aims to provide a comprehensive summary of the existing evidence regarding the impact of adverse early life exposures on IBS during pregnancy, intrapartum, and neonatal period. By consolidating this knowledge, the review enhances our understanding of the direct and indirect mechanisms underlying early life-related IBS and offers new insights and research directions from childhood to adulthood.

## 1. Introduction

Irritable bowel syndrome (IBS) is a prevalent and chronic gastrointestinal disease that affects individuals of various genders and age groups, characterized by recurring symptoms (1). The pathogenesis of IBS involves several factors, including visceral hypersensitivity, small intestinal bacterial overgrowth, intestinal dysbiosis, gut immune dysregulation, dietary intolerance, alterations in the gut-brain axis, and stress, among others. However, the precise mechanisms underlying IBS remain incompletely understood.

In the 1980s, D. Barker introduced the “Developmental Origins of Health and Disease” hypothesis (2), revolutionizing chronic disease studies by highlighting the importance of early life stages. Subsequently, numerous studies have demonstrated that early life exposures can increase the risk of metabolic, mental, cardiac, and chronic intestinal diseases in offspring (3–5).

Recent evidence has also revealed that the uterus is not a sterile environment (6), and gut microbiota composition in early life is less stable than in adults. Factors such as antibiotic use and dietary intake can disrupt the gut microbiota composition. Considering the crucial role of microbiota in intestinal growth and the development of IBS, it is evident that early life exposures can influence IBS by altering the initial gut microbiota composition.

**Figure 1:**
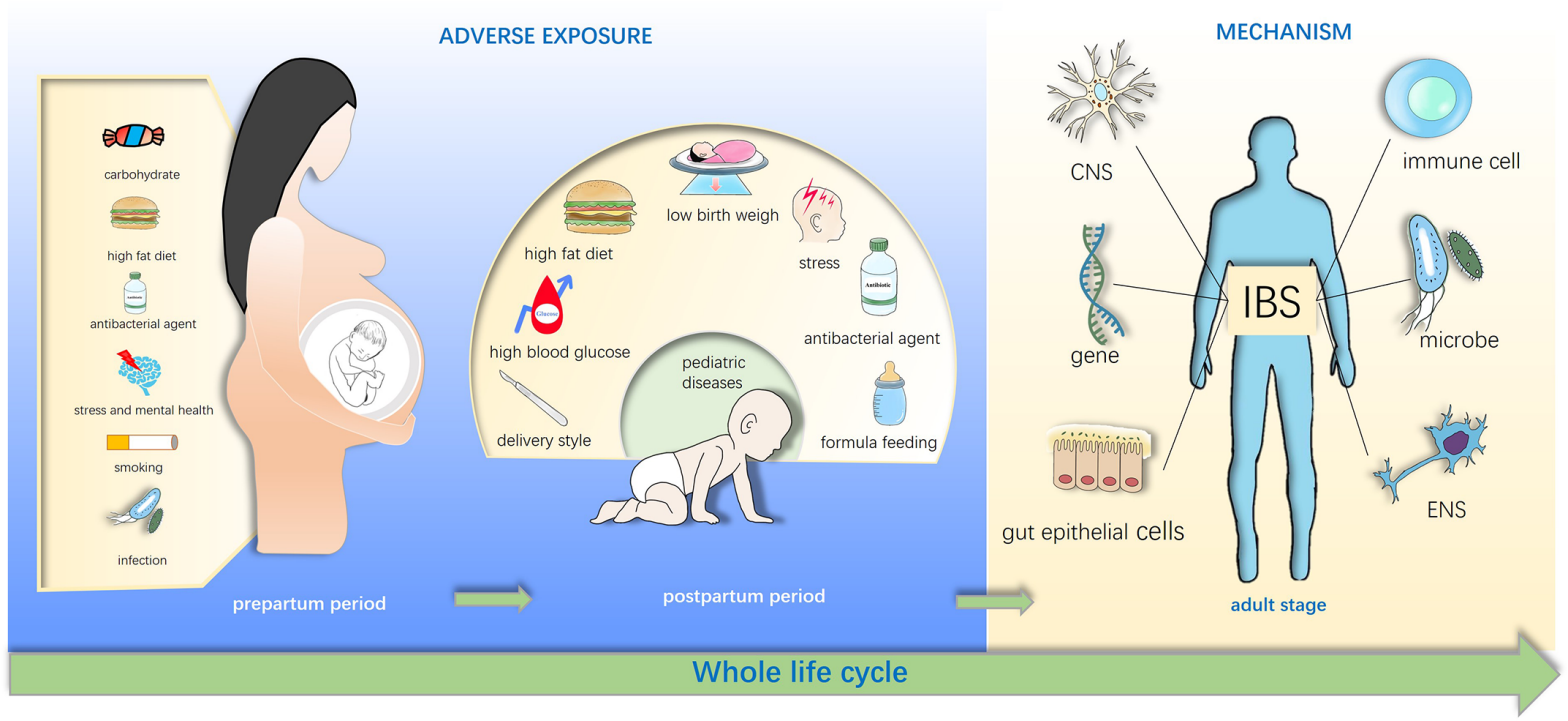
Early life adverse exposures might contribute to irritable bowel syndrome in adulthood. Adverse exposures in early life disturb the gut microbiota of infants and influence the brain-gut-microbiota axis, and these exposures include maternal diet exposure such as high fat diet, stress, drug use in pregnancy, cesarean section (C-section) and low birth weight, formula feeding of infants. These effects may directly or indirectly increase susceptibility of irritable bowel syndrome (IBS) in the childhood and even adulthood.

Furthermore, animal studies have shown that early-life exposure to maternal over-nutrition and antibiotics can cause dysbiosis, affecting offspring cognition and behavior (7, 8).While many studies indicate that early-life factors can affect offspring temporarily or long-term (9, 10) (Figure 1), the ability to prevent IBS is impeded by a lack of understanding of the intricate interplay between environmental factors and the disease.

Therefore, this review aims to summarize the maternal, intrapartum, and neonatal adverse exposures directly or indirectly related to the development of IBS (Table 1) and explore possible mechanisms. By doing so, this review will offer new insights into the prevention and treatment of IBS.

**Table 1:** Summary of early life adverse exposures affected directly in irritable bowel syndrome.

Adverse exposures		Authors	Publication year	Study type	Study populution	Outcome
High fat diet in offspring		Zhu et al. (11)	2014	Cross-sectional study	Chinese children in grades 1 through 6	Eating fried food is a risk factor for IBS
Stress exposure	NMS	Riba et al. (12)	2018	Experimental study	/	/
		Yi et al. (13)	2017	Experimental study	/	/
		Tang et al. (14)	2017	Experimental study	/	/
		O'Mahony et al. (15)	2009	Experimental study	/	/
	Early life adverse experiences	Park et al. (16)	2016	Case-control study	IBS patients and health controls	Various types of EALs are significant predictor of IBS
		Bradford et al. (17)	2012	Case-control study	IBS patients and health controls	Various types of EALs are associated with the development of IBS
		Ju et al. (18)	2020	Case-control study	IBS Patients and health controls	A greater number of EALs and higher perceived trauma severity were associated with increased odds of IBS
		Rahal et al. (19)	2020	Case-control study	IBS Patients and health controls	Fear improved prediction of IBS over the total number of EALs
Adverse perinatal period factors and formula feeding	C-section	Waehrens et al. (20)	2018	Cohort study	A national cohort of persons who were born in Sweden	Significant risk factors for IBS (caesarean, low birth weight, being second in birth orderfoetal growth ≥1 SD, young maternal age (<20 years), maternal marital status (divorced/widowed), maternal education of 10–11 years, maternal education of 12–14 years, parental history of IBS, parental history of anxiety, parental history of depression
	Lower birth weight	Waehrens et al. (20)	2018	Cohort study	As above	As above
		Raslau et al. (21)	2016	Case-control study	IBS Patients and health controls	Lower birth weight increased the odds for IBS
	Formula feeding	Koloski et al. (22)	2005	Case-control study	A random population sample from Sydney, Australia	Development of IBS was associated with childhood factors-a shorter duration of breastfeeding
Pediatric diseases related to IBS	Acute gastroenteritis	Cremon et al. (23)	2014	Cohort study	1,811 patients (primarily children) who were infected with foodborne Salmonella enteritidis in Bologna, Italy in 1994	The prevalence of IBS was higher in individuals exposed Salmonella as children than in controls
		Thabane et al. (24)	2010	Cohort study	Children exposed to domestic water contaminated with E. coli 0157:H7 and Campylobacter in Walkerton, Ontario, May 2000	Acute bacterial gastroenteritis is associated with subsequent IBS in children as in adults
	Functional constipation	Khan et al. (25)	2007	Case-control study	Pediatric FCC patients and health control	Childhood constipation appears to be a predictor of IBS in adulthood
	Urinary tract infection	Tan et al. (26)	2018	Cohort study	UTI infants and health controls	Infants with UTI had higher risks of childhood IBS
	Atopic dermatitis	Tsai et al. (27)	2018	Cohort study	AD children and health controls	AD children had a greater risk of developing IBS
	Food allergy/intolerance	Mansueto et al. (28)	2015	Experimental study	/	/
	Childhood physical/emotional trauma	Bradford et al. (17)	2012	Case-control study	294 IBS patients and 435 controls	EALs had an independent association with IBS
	Childhood abdominal pain	Howell et al. (29)	2005	Cohort study	1972 birth cohort (Dunedin, NZ)	CAP can progress to adult IBS in some children
	Asthma	Sjolund et al. (30)	2021	Cohort study	Children born in Sweden from 1994 through 1996	Asthma is positively associated with IBS

NMS, Neonatal maternal separation; C-section, cesarean-section; EALs, early adverse life events; FCC, functional childhood constipation; UTI, urinary tract infection; AD, atopic dermatitis; CAP, childhood abdominal pain.

## 2. Adverse exposures in early life and IBS

### 2.1. Maternal adverse factors

#### 2.1.1. Maternal inappropriate carbohydrate intake

Carbohydrates, found in diverse foods, are humans’ primary energy source. They can be classified into monosaccharides, disaccharides, and polysaccharides. Different dietary patterns can lead to variations in the composition of gut microbiota in offspring, and early-life microbiota alterations have been shown to influence susceptibility to diseases later in life (31). A cohort study conducted in Norway investigated 60 pregnant women and examined their dietary habits during pregnancy. Four days after delivery, the intestinal microbiota of these women was analyzed. The study found that increased sugar consumption was associated with a higher *Actinobacteria*/*Firmicutes* ratio (32). Another Australian pregnancy cohort study published in 2023 indicated a relationship between sugar consumption and decreased maternal microbial Shannon diversity (33). Conversely, reducing carbohydrate intake has been associated with an increased abundance of beneficial bacteria in the intestines, such as *Roseburia* spp., *Bifidobacterium* spp., and *Eubacterium rectale* (34). It has been established that there is an increase in pro-inflammatory bacteria in the gut of patients with irritable bowel syndrome (35). In 2021, Laura A. Bolte published high-quality evidence pointing out that the diet pattern is closely related to the composition of the microbiota and inflammation markers in human. Specifically, a high intake of carbohydrates has been shown to dramatically increase the presence of mucolytic bacteria and bacteria associated with energy harvesting, thereby promoting inflammation and increased gut permeability. Furthermore, long-term carbohydrate intake has been associated with higher quinone synthesis, which can also trigger intestinal inflammation (36). These studies have revealed the relationship between IBS, pro-inflammatory microbiota, and high sugar intake in adults. However, further evidence is needed to verify similar results in the early stages of life.

Carbohydrate intake has been closely linked to conditions like diabetes and obesity, as excessive consumption can lead to elevated blood glucose levels. The incidence of diabetes has been increasing alongside economic development. Interestingly, the incidence of IBS has also been on the rise in recent years. Specifically, a diet high in glucose and fructose was found to decrease the abundance of Bacteroidetes while increasing the levels of *Proteobacteria* and *Akkermansia muciniphila* in mice (37). These findings align with alterations observed in IBS patients and animal models (38, 39).

Interestingly, the offspring of the female rats in the diabetic model group had worse intestinal maturation when compared with those in the healthy control group. Specifically, the offspring from the diabetic model displayed thin intestinal mucosa and irregular arrangement of intestinal cells by the 10th day. As they reached the 45th day, the intestinal glands showed degeneration, and there was a further reduction in goblet cells (40). Furthermore, reports suggest that damage to the enteric nervous system (ENS) is involved in gastrointestinal motility changes in individuals with diabetes. This damage is believed to be linked to various factors, including enhanced apoptosis, oxidative stress, advanced glycation end products, alterations in intestinal muscle contractility, and brain-gut interactions. However, it is worth noting that these mechanisms are rarely discussed in the context of early life research (41).

Currently, there is no direct evidence demonstrating that a high-glycemic diet early in life promotes the occurrence of IBS (42). It is worth noting that the influence of maternal high sugar diet and maternal high blood glucose on IBS in offspring has not received adequate attention. Further research is needed to explore the potential effects of maternal high sugar diet and maternal high blood glucose levels on the development of IBS in offspring.

#### 2.1.2. Maternal high-fat diet

Increasing evidence links a high-fat diet to a heightened risk of inflammatory bowel disease. Shared pathogenesis between IBD and IBS indicates overlapping risk factors. Some researchers even propose that IBS represents the pre-IBD period (43, 44). In animal study, long-term consumption of a high-fat and high-sugar diet has already been established as a risk factor for pre-IBD (45). Experiments of mice have shown that interventions with a high-fat diet before, throughout, and after gestation can significantly alter the composition of the microbiota. Offspring from mice subjected to high-fat diet interventions exhibited increased levels of *Lachnospiraceae* and *Bacteroides*, and decreased levels of *Lactobacillus*, *Allobaculum*, and *Prevotella*. Importantly, these changes were not entirely consistent with alterations in the maternal microbiota. Furthermore, the effects of a high-fat diet could persist until adulthood in mice study (46). These changes in the microbiota caused by a high-fat diet create an opportunity for IBS. This hypothesis was supported by a previous cross-sectional study, which found a direct link between a high-fat diet in early life and the presence of IBS in children aged 8–13 years. The authors of the study suggested that dietary changes should be considered to prevent IBS in early life (11). Latest findings suggest adults with IBS consume more high-fat diets than those without the condition (47). Unfortunately, this study did not explore whether high-fat dietary habits were formed in the early life stage. Therefore, more research is needed to investigate dietary habits in early life.

In-depth studies have shown that a high-fat diet can alter the expression of genes associated with colon structure and function during the gut development of two-week-old transgenic mice. Specifically, genes such as Abca1, Mgat4b, Id1, and Tpp1 were found to be affected by the high-fat diet. Further analysis using a clustered image map revealed that the high-fat diet induced changes in the function of the colon by regulating these genes in these mice, which the microbiota may influence in some way (48). Moreover, it has been reported that a maternal high-fat diet could increase anxiety-like behavior in female macaque offspring. This finding is significant given that psychological factors have been established as important pathogenesis mechanisms in IBS (49).

#### 2.1.3. Maternal antibacterial agent exposure

Antibiotic exposure has been shown to be a risk factor for IBS in adulthood in human (50). However, there is limited direct evidence indicating that maternal antibiotic exposure can trigger the occurrence of IBS in adulthood.

The gut microbiota of infants is highly susceptible to the effects of antibiotics. Disruptions in microbiota composition and microbial colonization can significantly affect human health (51). One of the most notable changes in the gut microbiota following antibiotic exposure is the enrichment of three genera: *Bacteroides*, *Peptostreptococci*, and *Enterobacteria*, while the abundance of the *Bacteroidetes* phylum is decreased (52). Additionally, in a rat study, beneficial bacteria like *Lactobacillus* are reduced, and there is an increase in the relative abundance of pathogenic bacteria such as *Enterobacter, Shigella sonnei, Enterococcus hormaechei, and Acinetobacter sp.* (53). A European infants study observed that maternal antibiotic use during the perinatal and/or breastfeeding periods could decrease the number of *Bacteroides*, and antibiotic use in newborns could shape the microbiota composition by increasing the proportion of *Enterobacteria* (54), which is shared by patients with IBS. These changes in gut flora mentioned above can increase the risk of intestinal diseases, including IBS, by modulating the intestinal microbiota. However, a case-control study published in 2016 reported that early-life antibiotic use is not a risk factor for IBS (21). Long-term assessments of antibiotic effects on gut flora and large-scale retrospective studies are still needed to investigate the relationship between early-life antibiotic exposure and the incidence of IBS.

#### 2.1.4. Maternal stress

Prenatal maternal stress treatment can inhibit intestinal development in offspring mice at 3 weeks of age, impair their intestinal barrier function, and induce low-grade inflammation in the gut (55) Beyond directly influencing the gastrointestinal development of the offspring, maternal stress can also disrupt the neuroimmune network in the offspring. The fetal immune system and central nervous system are very sensitive to external disturbances during pregnancy, so stress in early life can enhance the function of HPA axis, increase systemic immune response and disturb intestinal microbiota in offspring (15). The early immune system change can persist throughout the whole life, and the maternal immune functional disturbances caused by stress can be passed to the next generation, which means that the ability of offspring to absorb maternal immunoglobulin is impaired and immune system is depressed (56, 57). Similarly, research has begun to uncover how these disturbances may affect the gut environment of the offspring. In 2023, a study revealed the impact of maternal stress on the offspring's gut microbiota by examining the fecal microbiota of infants. The study found that maternal psychological stress led to a decrease in offspring's gut microbial alpha diversity and a reduction in the number of probiotic like *Bifidobacterium*. Therefore, maternal stress might cause the occurrence of IBS by triggering gut microbiota disorder in the offspring (58). Animal experiments have also found that prenatal stress leads to a reduction in the number of *Bifidobacteria* and *Lactobacilli* in the feces of young monkeys (59). Furthermore, a rat study has shown that prenatal maternal stress can lead to visceral hypersensitivity in the offspring, and this change may be mediated by the upregulation of cystathionine-β-synthase and Nav1.7 expression. Animal studies have also reported that miR-485/ASIC1 signaling and BDNF expression are associated with the occurrence of visceral hypersensitivity in offspring following prenatal stress (60, 61). Therefore, stress during pregnancy could also increase the risk of offspring developing IBS through a mechanism of visceral hypersensitivity (62).

#### 2.1.5. Maternal smoking

Currently, there is no direct evidence that maternal smoking leads to an increased risk of IBS in offspring. However, human studies have found that maternal smoking exposure during pregnancy is associated with DNA methylation in offspring peripheral blood, unfortunately, these methylation sites were not found to be associated with IBS (63). Further research emphasizes the multifaceted impact of maternal smoking on offspring. Smoking during pregnancy leads to a decrease in fecal microbiota diversity in newborns, and an increase in colonization by *Enterobacteriaceae*. At 6 months, these infants have a higher abundance of *Bacteroides* and *Staphylococcus* in their feces (64).Studies on autism have shown that maternal smoking can cause the onset of autism by altering the gut microbiota and acting through the gut-brain axis, as well as shaping early brain development (65, 66). However, no studies have proven that maternal smoking promotes the occurrence of IBS through these mechanisms.

#### 2.1.6. Maternal mental health

Maternal mental health may have an impact on the onset of IBS in offspring. Familial clustering of IBS has been observed, and in addition to genetic factors, sociological learning can also affect the prevalence of IBS in offspring. Early-life sociological learning primarily comes from parents. Studies have pointed out that the offspring of mothers with IBS are more likely to be troubled by gastrointestinal symptoms, and both mothers and children are more likely to experience anxiety and depression. This could be related to the intergenerational transmission of psychological disorders such as anxiety and depression (67). Therefore, maternal mental health is extremely important. Moreover, research on infant gut microbiota suggests that maternal anxiety and depression can affect the composition of the offspring's gut microbiota, reducing the number of probiotics, changing the levels of cytokines in the body, and thereby increasing the likelihood of offspring suffering from IBS through brain-gut axis mechanisms (58). However, there have also been contradictory conclusions. Some studies have confirmed a connection between maternal anxiety and a decrease in pro-inflammatory bacteria like Proteobacteria, in infants, while other studies have proposed opposite conclusions. The authors believe this may be due to differences in fecal microbiota detection methods and ways of measuring maternal anxiety (59).

#### 2.1.7. Maternal infection

Current evidence suggests a strong association between maternal infection and the onset of neuropsychiatric disease in offspring (68), but there isn't enough evidence to indicate that maternal infection during pregnancy increases the risk of IBS in offspring. More animal experiments and cohort studies are needed in the future to elucidate the relationship between infection during pregnancy and IBS.

### 3.1. Postpartum adverse factors

#### 3.1.1. Neonatal high blood glucose

Type 1 diabetes (T1D) typically occurs in early life stages. Although the mechanisms of T1D are diverse, it is useful for understanding the connection between high blood glucose and IBS in early life. A Finnish cohort study analyzed fecal microbiota compositions from pediatric T1D patients. The study observed a decrease in alpha diversity and an increase in the presence of *Ruminococcus gnavus* and *Streptococcus infantarius* in T1D patients. These two strains are known to be pathogenic bacteria that can disrupt the intestinal barrier and promote gut inflammation. Additionally, further investigation found elevated levels of human β-defensin 2 (hBD2) in samples from T1D patients. hBD2 is an antimicrobial peptide produced by epithelial cells to defend against pathogens (69). It has been previously established that IBS is characterized by low-grade inflammation in the gut (70). Therefore, the evidence mentioned above reveals the relationship between increased inflammation in the gut and metabolic disturbances in early life.

Glucagon-like peptide 1 (GLP-1) is a molecule that plays a significant role in regulating blood glucose levels. It is produced by enteroendocrine L-cells in the intestine. It has been established that carbohydrates in the lumen of the intestine stimulate the secretion of GLP-1, suppressing gastrointestinal motility in human (71, 72). While studies have indicated that GLP-1 analogs can alleviate abdominal pain in IBS patients, the exact role of GLP-1 in IBS physiology is still not fully understood. An animal study suggested that increased levels of GLP-1 are involved in the mechanism of IBS-C (73). However, a different human study found decreased levels of GLP-1 in the blood of IBS-C patients (74). Furthermore, a human study published in 2015 demonstrated that maternal high blood glucose levels could lead to decreased GLP-1 levels in offspring (75). From this, we can infer that maternal dietary patterns that result in high blood glucose may represent a new risk factor for the development of IBS in offspring.

#### 3.1.2. High-fat diet in offspring

It is now understood that high-fat diets contribute to obesity and have been established as risk factors for cardiovascular and metabolic diseases. In addition, a cross-sectional study conducted in China demonstrated that a high-fat diet is also a risk factor for IBS in children (11). Interestingly, obesity has been extensively linked to the development of IBS. Studies have shown that obese adults are more likely to be diagnosed with IBS, and similar findings have also been reported in children (76). Dysbiosis, or an imbalance in the gut microbiota, is one of the main mechanisms associated with IBS. Interestingly, similar alterations in gut microbiota have been observed in obese individuals (77). Moreover, further evidence suggests possible connections between the microbiota profiles associated with IBS and those induced by a high-fat diet in early life (78, 79).

Currently, there is no direct evidence indicating that a high-fat diet in early life leads to an increased incidence of IBS in adulthood. An animal study showed that a high-fat diet given to mice from weaning until 6 weeks of age significantly reduced the relative abundance of the *Muribaculaceae* family in the mouse gut microbiota by the age of 14 weeks. *Muribaculaceae* is associated with the production of propionate, a short-chain fatty acid (80). Other animal studies have pointed out that a high-fat diet in juvenile mice reduces the number of *Bifidobacterium* and *Akkermansia* in the gut, and promotes the increase of pro-inflammatory bacteria like *Dorea* (81). Importantly, a study involving juvenile high-fat diet intervention demonstrated increased plasma insulin levels and decreased insulin sensitivity (82). These metabolic changes may interact with GLP-1 secretion, as discussed earlier. Furthermore, disruption of the enteric epithelial barrier and increased intestinal permeability have been observed in patients with IBS (83). Early-life high-fat diet intake might, based on evidence, predispose to IBS by damaging the gut barrier and enhancing intestinal permeability. Toll-like receptor 4 (TLR4) is a member of the pattern recognition receptor family and plays a role in the inflammatory process in animal models of obesity. Recent studies have indicated that mice receiving a high-fat diet for 16 weeks starting from 3 weeks old exhibited visceral hypersensitivity. This outcome could be attributed to the increased expression of TLR4 protein in both the central and peripheral nervous systems (84).

#### 3.1.3. Neonatal stress exposure

According to the Rome IV criteria, IBS is considered a disease related to the gut-brain axis, where stress and psychological factors play a significant role in its pathogenesis. Clinical studies have shown a strong correlation between early life adverse experiences and the development of IBS (18). A case-control study supported that early-life emotional abuse increases the risk of developing IBS in adulthood. Additionally, another study identified household mental illness as the strongest predictor for IBS in adulthood among various early-life adverse events (16, 17). Further research has suggested that a sense of fear and dissociation following early-life trauma can predict the occurrence of IBS later in life (19).

Neonatal maternal separation (NMS) has become a widely accepted experimental model for studying IBS, and it has provided insights into the mechanisms related to stress in IBS development (12). Animal experiments using this model have demonstrated that NMS can induce visceral hypersensitivity and abnormal behavior in rats from early life to adulthood (13). The underlying mechanism for these effects involves increased Toll-like receptor 4 (TLR4) expression in microglial cells (14). Regarding visceral hyperalgesia, nerve growth factor (NGF) is crucial in mediating neuronal plasticity (85). In an animal experiment, male Wistar rats were subjected to NMS for 3 h per day starting from the second to fourteenth days after birth. When sacrificed at the 12th week, these rats exhibited colon mast cell hyperplasia and increased neurokinin 1 (NK1) receptor expression in the spinal cord. It has been established that substance P, which binds to NK1 receptors, can mediate pain transmission and regulates pain responses. During maternal separation, anti-NGF antibody treatment in NMS rats reduced enteric nerve synapses formation and mast cell counts, implying NGF's role in early-life stress-induced IBS development (86).

Early immune system disturbance and dysfunction of the HPA axis have been closely associated with IBS and can persist throughout an individual's life. male NMS rats have been observed to exhibit increased corticosterone levels, enhanced HPA axis activity, and abnormal behaviors (15). In NMS rats, the activation of metabotropic glutamate receptor-7 has been shown to reduce visceral hypersensitivity by modulating immune responses (87).

The intestinal mucosa contains a large number of immune cells and is vital to the functioning of the immune system. Animal studies have shown that at the age of 14 days, the colonic mucosa of neonatal maternal separation (NMS) rats showed an increase in mast cells compared to the control group, a phenomenon that persisted at 12 weeks. Simultaneously, the experiment found that at 14 days and 12 weeks, the expression of NGF in the mucosal and muscular layers of the NMS rat colon was significantly increased compared to the control group, consistent with changes in intestinal hypersensitivity in both groups (88). Other study has also revealed persistent hypersensitivity function of secretomotor neurons and upregulation of acetylcholine activity in pig offspring on the 15th day after weaning. Additionally, more enteric neurons have been observed, suggesting a potential link to IBS (89). These findings provide a foundation for understanding the relationship between early life stress and the development of IBS.

Psychological therapy has emerged as an important approach to managing stress-related disorders, including IBS. Studies have shown that cognitive behavioral therapy and hypnotherapy have effectively treated children and adults with IBS (90). These therapeutic approaches offer promising directions for future research and highlight the significant role of psychological interventions in managing IBS symptoms.

#### 3.1.4. Neonatal formula feeding

Neonatal feeding modalities encompass breastfeeding, formula feeding, and mixed feeding. Recent research has shed light on the benefits of breastfeeding in growth programming (91). Breastfeeding during the initial three months has been linked to reduced infant functional constipation and favorable modulation of gut microbiota, influencing overall health (92). Evidence suggests that breastfed infants exhibit increased levels of probiotic species, such as *Bifidobacterium*, in their feces, often more than double that of formula-fed infants. A follow-up study published in 2023 indicated that formula feeding at 12 months of age was associated with a decrease in Verrucomicrobiota compared to breastfeeding (93). Conversely, formula-fed infants showed a decrease in *Bifidobacterium* levels and increased *Bacteroides* abundance (94, 95). Furthermore, formula-fed infants exhibited an overrepresentation of *C. difficile*, and a recent experiment on neonatal piglets demonstrated that formula-feeding predisposed them to *C. difficile* gut infection (96). Additionally, the effects of combining breast milk with formula feeding on the microbiota were more similar to exclusive formula feeding rather than exclusive breastfeeding (97).

Interestingly, prolonged infant breastfeeding has enhanced their resilience against IBS in adulthood (22). This effect is believed to be associated with the modulation of Th1 and Th2 immune responses. In individuals with IBS, the immune response shifts towards Th2 cells (98). Breastfeeding has been found to promote the development of a Th1 response in human (22). Additionally, human milk oligosaccharides have been shown to benefit by modulating cell signaling, gut microbiota composition and reducing mucosal invasion (99). In contrast, exclusive formula feeding can alter gut microbiota and increase susceptibility to gastrointestinal diseases (100). Studies have indicated that formula feeding can lead to changes in gastrointestinal morphology, microbial abundance, intestinal barrier proteins (such as vascular endothelial cadherin), and interleukin-10 (IL-10) production (101, 102). These alterations may result in reduced immune education and increased inflammatory processes. Improving feeding patterns can enhance immunity, reduce inflammation, and help prevent allergic and infectious diseases. Importantly, evidence suggests that immune impairments can have long-term health consequences. A study involving five European centers found that feeding patterns have a sustained influence on intestinal microbiota, which persists even after weaning (103).

Therefore, the choice of feeding method can have a long-term impact on the gut microbiota, immune system, and intestinal barrier of offspring. Optimal feeding methods play a crucial role in preventing the development of IBS from childhood to adulthood.

#### 3.1.5. Neonatal antibiotic use

Studies have consistently shown that antibiotic exposure in adults and children increases the risk of abdominal symptoms, particularly abdominal pain (104). The changes in gut microbiota caused by early-life antibiotic use have been linked to the development of abdominal pain. For example, a Swedish study involving 2,732 12-year-old children found that antibiotic use during the first 2 years of life was associated with an increased risk of abdominal pain, especially in girls (105). Additionally, long-term or broad-spectrum antibiotic use between the ages of 9 and 12 was found to contribute to the risk of abdominal pain (106). Since the diagnosis of IBS is based on abdominal symptoms, it is noteworthy that disorders of gut-brain interaction in early life have been identified as independent predictors of IBS in adulthood (25). This suggests that many individuals experience IBS symptoms (such as altered bowel habits, changes in frequency of bowel movements, abdominal cramps, decreased appetite, and/or early satiety, gas and bloating) from early life into adulthood (107). Further research is needed to explore the long-term implications of these findings and provide a more comprehensive understanding of the underlying mechanisms.

Current research suggests that early-life antibiotic use directly impacts intestinal epithelial cells. In mouse experiments, early-life antibiotic exposure accelerated intestinal epithelial maturation, reducing permeability and the count of vacuolated enterocytes (108). These changes may be attributed to the stage of intestinal epithelium development during early life. In contrast, antibiotic use in adults is often associated with increased intestinal permeability, which may reflect differences in the developmental stage of the intestinal epithelium. The decrease in vacuolated enterocytes resulting from early-life antibiotic use can affect the absorption of breast milk and potentially diminish the protective effects associated with breastfeeding.

Indeed, antibiotic use in early life, including the first two years of childhood, has been associated with an increased risk of various allergic diseases, including food allergies (109). Allergic diseases, in turn, have been linked to an increased risk of abdominal pain (110). Food allergy has also been considered one of the possible causes of IBS (28). Furthermore, besides clinical use, antimicrobial substances like triclosan and triclocarban, commonly found in household and personal care products, can also disrupt the gut microbiota composition. Studies have shown that increased levels of triclosan are associated with decreased abundance of *Bacteroides fragilis* and enrichment of the *Proteobacteria* phylum in infants (111). Dysbiosis of the gut microbiota has been implicated in the development of functional gastrointestinal disorders and can potentially affect cognitive function (112). Therefore, it can be inferred that early-life antibiotic use, including exposure to antimicrobials, may negatively influence the gut microbiota of offspring and contribute to the onset of IBS in adulthood. However, more extensive research is still needed to better understand the relationship between antibiotic use, gut microbiota, and the development of IBS, especially through large-scale studies and data analysis.

#### 3.1.6. Low birth weight

Low birth weight has been identified as another risk factor for IBS (20, 21). It is unclear what are causes of low birth weight (113). It has been suggested that the increased risk of IBS associated with low birth weight may be attributed to the immaturity of intestinal motor function (21, 114). Moreover, very low birth weight infants have been found to exhibit abnormal patterns of intestinal microbiota colonization, characterized by reduced population diversity and an increase in bacterial and fungal pathogens such as *Escherichia coli, Enterococcus sp., Klebsiella pneumoniae, Candida spp., and Clavispora spp*. These findings support the role of altered microbiota in the pathogenesis of IBS, as discussed earlier (115, 116).

#### 3.1.7. Pediatric diseases related to IBS

Indeed, several pediatric diseases have been discovered to have notable correlations with IBS, highlighting a complex and multi-faceted etiological landscape. Functional constipation and Salmonella gastroenteritis in early life, for instance, have been identified as risk factors for IBS in adulthood (23). Furthermore, there is emerging evidence of a cross-talk between the urinary and intestinal systems, with studies supporting the idea that genitourinary disorders can contribute to gastrointestinal disorders (117, 118). Infants with urinary infections have been found to have a higher risk of developing IBS in childhood (26), possibly due to their shared embryonic origin and peripheral nerve connections (119).

Childhood physical or emotional trauma is a risk factor for developing IBS in adulthood, with studies indicating that early general trauma, physical punishment, emotional abuse, and sexual events are strong predictors for the onset of IBS in adulthood (17). Additionally, post-traumatic stress disorder in adult has also been confirmed as an adverse exposure for IBS (120), but the research did not focus on the early life period. Evidence suggests that fear emotions generated by early physical or emotional trauma play a critical role in the pathophysiology of IBS (19). A prospective study published in 2005 pointed out that some childhood abdominal pain can develop into IBS in adults, though the specific mechanisms remain elusive (29). In contrast, other research has found that early life recurrent abdominal pain may not be connected with IBS in adolescence, leaving unresolved questions regarding the relationship between childhood abdominal pain and adult IBS (121).

From the perspective of IBS research, there's a complex interplay between the syndrome and associated allergic diseases. Sudies have found that children with a history of atopic dermatitis (AD) are more prone to IBS (27). Genetic variations and local mucosal immune function have been implicated in both atopy and IBS (122, 123). Children with AD often experience food allergies closely related to mast cell activation, and these children have a higher risk of gastrointestinal dysfunction. Although the exact mechanisms are not yet fully understood, immune responses in the small bowel have been proposed as a potential link between food allergy and IBS in adults (124). Exploring the immune changes in the small bowel in children and their relationship to the development of IBS in adulthood holds promise for future research in this area.

Diving deeper into allergy-related diseases, a recent study have also drawn connections between childhood asthma and the onset of IBS by age 16. The same research pointed out that children who had eczema at 1–2 years of age might have an increased trend to develop IBS at 16 years of age, but unfortunately, the data did not show a significant statistical difference. Food hypersensitivity is generally associated with IBS onset at age 16, but the data did not indicate whether such a link exists in early life (30).

### 4.1. Delivery style

Microbiota is shared between the gut and vagina, and the mode of delivery significantly affects the microbiota in newborns. Several studies have demonstrated that caesarean section (C-section) delivery increases the risk for IBS (125). The mechanism underlying this association may be linked to the composition of the microbiota. Vaginally delivered and exclusively breastfed infants exhibit the “best” gut microbiota composition, characterized by a higher abundance of beneficial bacteria such as *Bifidobacteria* and *Bacteroides*, and a lower abundance of potentially harmful bacteria like *Escherichia coli (E. coli) and Clostridium difficile* (125). In contrast, infants born via C-section seem to have a lower abundance and diversity of gut microbiota and decreased and delayed colonization of *Bifidobacterium spp.* and *Bacteroides spp.* Instead, they tend to have increased levels of *C. difficile* and typical skin bacteria like *Staphylococcus, Corynebacterium, and Propionibacterium spp* (126). Similar results have been found in a large-scale study from the Netherlands, where C-section delivery was associated with lower amounts of *Bifidobacteria* and *Bacteroides fragilis* (*B. fragilis*)-group species and increased amounts of *E. coli* and *C. difficile* (125). Interestingly, emerging research suggests that the influence of maternal microbiota on the infant's microbiota is only significant in vaginally-delivered infants, and this influence is disturbed by C-section delivery (127). In addition, C-section delivery has been associated with a higher rate of acute gastroenteritis admission in children (128). It has been reported that a history of acute gastroenteritis in children is linked to an increased risk of IBS (24).

## 5. Conclusions

Lately, there has been increasing evidence showing that adverse exposures in early life can impact the development of functional gastrointestinal diseases, including IBS, in adulthood. These factors during early life stages can influence the risk of developing IBS later. Some factors, such as diet and stress, are relatively well-established, while others, including the use of antibiotics, delivery mode, and feeding patterns, still require long-term follow-up observations to gather sufficient evidence.

Proper diets and interventions with probiotics in early life have shown potential therapeutic prospects for preventing IBS. Therefore, understanding the relationship between adverse exposures in early life and IBS can open up a new field for preventing IBS in adulthood. This knowledge can ultimately help reduce the disease's burden and contribute to improving population health and the rational use of medical resources.
